# Supplementary material for: The causal relationship between the human gut microbiota and pyogenic arthritis: a Mendelian randomization study
Source: Front Cell Infect Microbiol. 2024 Nov 26;14:1452480. doi: 10.3389/fcimb.2024.1452480 (PMC11629706; doi:10.3389/fcimb.2024.1452480)
Supplement: Supplementary file 1 [file DataSheet1.zip › mendelian test/ebi-a-GCST90027549.csv_leave_one_out.pdf]

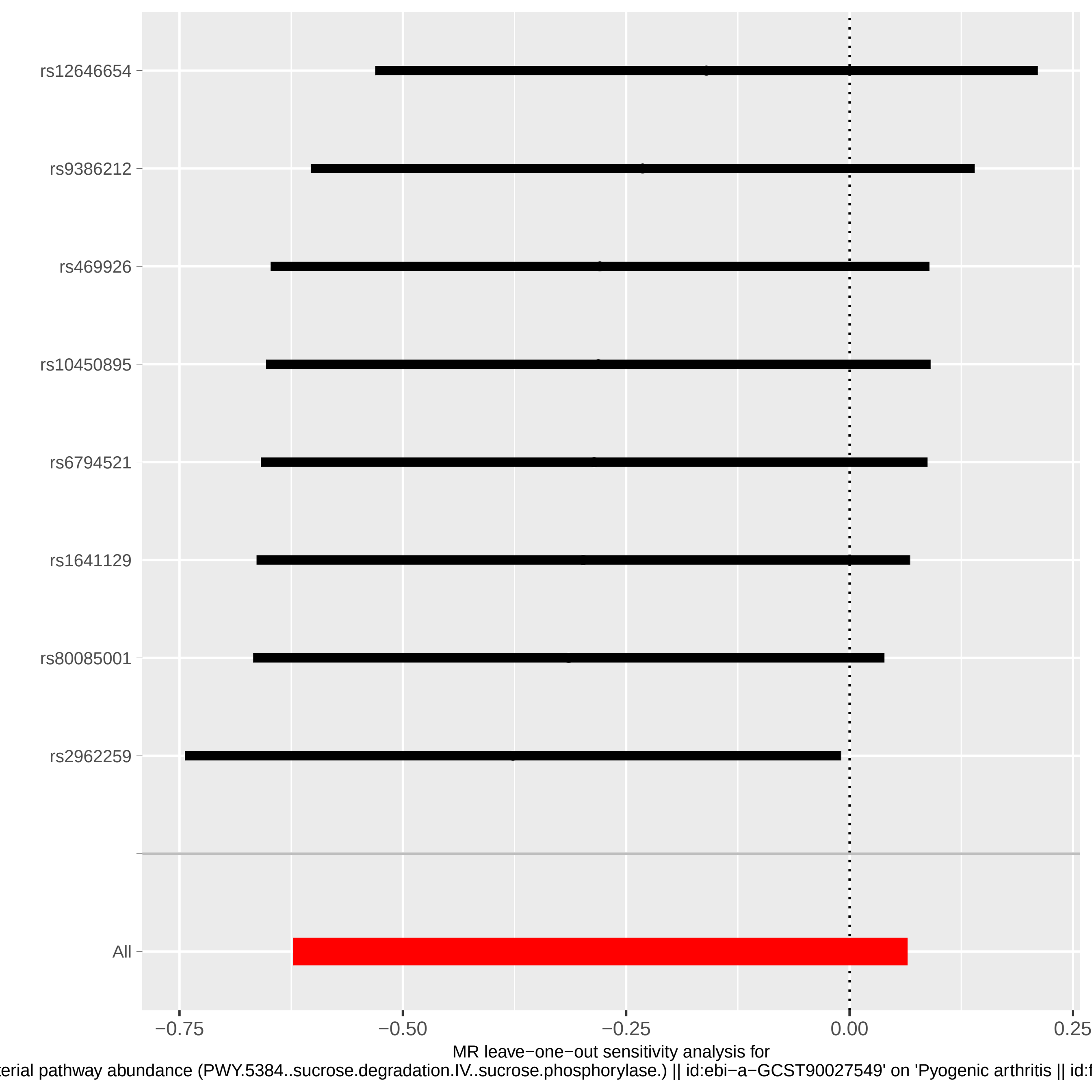

rs12646654
rs9386212
rs469926
rs10450895
rs6794521
rs1641129
rs80085001
rs2962259
All
−0.75
−0.50
−0.25
0.00
0.25
MR leave−one−out sensitivity analysis for
terial pathway abundance (PWY.5384..sucrose.degradation.IV..sucrose.phosphorylase.) || id:ebi−a−GCST90027549' on 'Pyogenic arthritis || id:f
